# Hydroxonium hydrate tris­(2,4,6-tri­amino-1,3,5-triazin-1-ium) bis­[bis­(pyri­dine-2,6-dicarboxyl­ato)cuprate(II)] pyridine-2,6-dicarboxylic acid hexa­hydrate

**DOI:** 10.1107/S1600536809000828

**Published:** 2009-01-14

**Authors:** Hossein Aghabozorg, Jafar Attar Gharamaleki, Marilyn M. Olmstead, Zohreh Derikvand, Shabnam Hooshmand

**Affiliations:** a Faculty of Chemistry, Tarbiat Moallem University, 49 Mofateh Avenue, Tehran, Iran; b Department of Chemistry, University of California, One Shields Avenue, Davis, CA 95616, USA; c Faculty of Science, Department of Chemistry, Islamic Azad University, Khorramabad Branch, Khorramabad, Iran; d Department of Chemistry, Ilam University, Ilam, Iran

## Abstract

The reaction of copper(II) nitrate hexa­hydrate with pyridine-2,6-dicarboxylic acid (pydcH_2_) and 2,4,6-triamino-1,3,5-triazine (melamine) in aqueous solution in a 1:2:2 molar ratio gave the title compound, (H_5_O_2_)(C_3_H_7_N_6_)_3_[Cu(C_7_H_3_NO_4_)_2_]_2_·C_7_H_5_NO_4_·6H_2_O. The hydroxonium hydrate (H_5_O_2_)^+^, also known as the Zundel cation, resides on a twofold rotation axis. The O—H distance is 1.274 (14) Å, the O⋯O distance is 2.518 (5) Å, and the O—H—O angle is 162 (8)°. One of the melamine H^+^ cations, the uncoordinated pydcH_2_, and two water mol­ecules also reside on crystallographic twofold axes. The Cu^II^ atom has a tetra­gonally distorted octa­hedral coordination environment. The structure features extensive hydrogen bonding, with 21 distinct inter­actions. There is also a centrosymmetric C=O⋯π inter­action with an O⋯centroid distance of 3.288 (3) Å. The structure is similar to a mixed-valence manganese(II/III) structure but shows inter­esting differences in the metal-atom coordination. One of the water molecules is equally disordered with respect to a twofold axis.

## Related literature

For related melamine salts, see: Aghabozorg, Aghajani & Sharif (2006 ▶); Aghabozorg, Attar Gharamaleki *et al.* (2008 ▶); Aghabozorg, Ghadermazi *et al.* (2008 ▶); Aghabozorg, Manteghi & Sheshmani (2008 ▶); Aghabozorg, Zabihi *et al.* (2006 ▶); Agha­­jani *et al.* (2006 ▶); Perpétuo & Janczak (2006 ▶); Sharif *et al.* (2006 ▶, 2007 ▶); Zhang & Chen (2005 ▶). For a nearly isostructural manganese(II/III) structure, see: Aghabozorg, Derikvand *et al*. (2008 ▶).
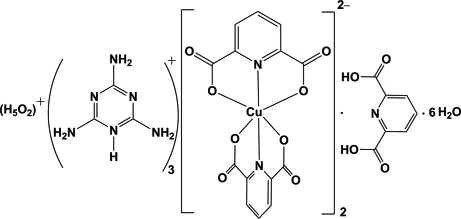


## Experimental

### 

#### Crystal data

(H_5_O_2_)(C_3_H_7_N_6_)_3_[Cu(C_7_H_3_NO_4_)_2_]_2_·C_7_H_5_NO_4_·6H_2_O
*M* *_r_* = 1481.19
Monoclinic, 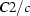

*a* = 27.575 (3) Å
*b* = 22.814 (3) Å
*c* = 9.8068 (12) Å
β = 108.327 (2)°
*V* = 5856.5 (13) Å^3^
*Z* = 4
Mo *K*α radiation
μ = 0.84 mm^−1^
*T* = 180 (2) K
0.35 × 0.28 × 0.02 mm

#### Data collection

Bruker SMART APEXII diffractometer
Absorption correction: multi-scan (*SADABS*; Sheldrick, 1996 ▶) *T*_min_ = 0.758, *T*_max_ = 0.983
26801 measured reflections
5309 independent reflections
3805 reflections with *I* > 2σ(*I*)
*R*_int_ = 0.051

#### Refinement

*R*[*F* ^2^ > 2σ(*F* ^2^)] = 0.035
*wR*(*F* ^2^) = 0.096
*S* = 1.03
5309 reflections
531 parameters
21 restraints
H atoms treated by a mixture of independent and constrained refinement
Δρ_max_ = 0.26 e Å^−3^
Δρ_min_ = −0.77 e Å^−3^

### 

Data collection: *APEX2* (Bruker, 2007 ▶); cell refinement: *SAINT* (Bruker, 2007 ▶); data reduction: *SAINT*; program(s) used to solve structure: *SHELXS97* (Sheldrick, 2008 ▶); program(s) used to refine structure: *SHELXL97* (Sheldrick, 2008 ▶); molecular graphics: *SHELXTL* (Sheldrick, 2008 ▶) and *Mercury* (Macrae *et al.*, 2006 ▶); software used to prepare material for publication: *SHELXL97*.

## Supplementary Material

Crystal structure: contains datablocks I, global. DOI: 10.1107/S1600536809000828/gw2056sup1.cif

Structure factors: contains datablocks I. DOI: 10.1107/S1600536809000828/gw2056Isup2.hkl

Additional supplementary materials:  crystallographic information; 3D view; checkCIF report

## Figures and Tables

**Table 1:** Hydrogen-bond geometry (Å, °)

*D*—H⋯*A*	*D*—H	H⋯*A*	*D*⋯*A*	*D*—H⋯*A*
N3—H3*A*⋯O6	0.87 (3)	1.903 (12)	2.766 (3)	168 (3)
N6—H6*A*⋯O4^i^	0.876 (13)	2.062 (17)	2.880 (4)	155 (3)
N6—H6*B*⋯O5	0.88 (3)	2.007 (12)	2.872 (3)	167 (3)
N7—H7*A*⋯N9^ii^	0.88 (3)	2.086 (11)	2.965 (3)	176 (3)
N7—H7*B*⋯O9^ii^	0.879 (12)	2.13 (3)	2.815 (3)	134 (3)
N8—H8*B*⋯O14	0.88 (3)	1.964 (11)	2.842 (3)	177 (3)
N10—H10*A*⋯O15	0.882 (12)	1.782 (12)	2.649 (6)	167 (2)
N11—H11*A*⋯O9^iii^	0.88 (3)	2.064 (11)	2.939 (3)	172 (3)
N12—H12*A*⋯N5^ii^	0.87 (3)	2.090 (12)	2.959 (4)	173 (3)
N12—H12*B*⋯O1	0.882 (12)	2.07 (2)	2.846 (3)	146 (3)
O10—H10*B*⋯O13	0.85 (3)	1.83 (3)	2.671 (3)	172 (4)
O11—H11*B*⋯O2^iv^	0.81 (3)	2.10 (3)	2.896 (3)	167 (4)
O12—H12*C*⋯O8^iii^	0.84 (3)	1.72 (3)	2.555 (3)	173 (4)
O12—H12*D*⋯O2	0.84 (3)	1.89 (4)	2.700 (3)	162 (5)
O12—H12*E*⋯O12^iv^	1.274 (14)	1.274 (14)	2.518 (5)	162 (8)
O13—H13*A*⋯O4^v^	0.84 (3)	2.04 (3)	2.870 (3)	172 (4)
O13—H13*B*⋯N4^ii^	0.84 (3)	2.066 (17)	2.876 (3)	162 (4)
O14—H14*A*⋯O6	0.84 (3)	1.831 (12)	2.662 (3)	172 (3)
O14—H14*B*⋯O3^v^	0.84 (3)	2.05 (4)	2.850 (3)	160 (4)
O15—H15*A*⋯O11^vi^	0.84 (3)	1.98 (6)	2.748 (6)	151 (11)
O15—H15*B*⋯O7^iii^	0.84 (3)	1.82 (2)	2.636 (5)	163 (7)

Symmetry codes: (i) 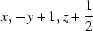; (ii) 
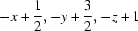
; (iii) 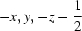; (iv) 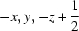; (v) 
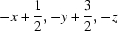
; (vi) 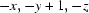.

**Fig. 1.**
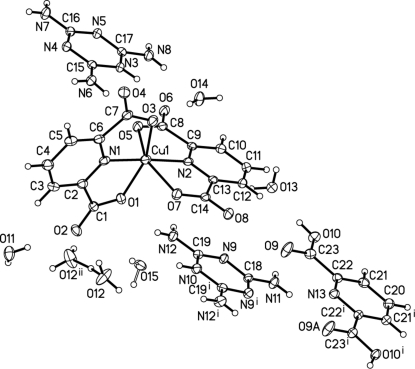
A view of the constituents of the title compound showing the atom numbering scheme. Displacement ellipsoids are drawn at the 30% probability level. Symmetry codes: (i) -x, y, -1/2 - z, (ii) -x, y, 1/2 - z.

**Fig. 2.**
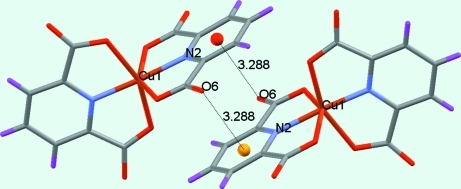
Centrosymmetrically related (symmetry code 1/2 - x, 3/2 - y, -z) C=O···π interaction between C=O groups and centroid of the N2/C9—C13) aromatic ring of pyridine-2,6-dicarboxylate. The distances between the O atom and the ring centroid is 3.288 (3) Å.

**Fig. 3.**
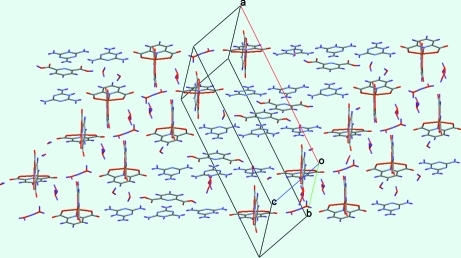
A packing diagram of the title compound. The space between layers of [Cu(pydc)2]2– is filled with a layer of (H5O2)+ and (melamineH)+ cations, pydcH2 and water molecules.

## supplementary crystallographic information

### Comment

We have previously reported a proton-transfer system using pyridine-2,6-dicarboxylic acid (pydcH_2_) and 2,4,6-triamino-1,3,5-triazine (melamine, also called tata), (melamineH)_2_(pydc) (Sharif *et al.*, 2006). We also reported some complexes of this system (Aghabozorg, Aghajani *et al.*, 2006; Aghajani *et al.*, 2006; Sharif *et al. *2007; Aghabozorg, Attar Gharamaleki *et al. *2008). In the title compound, melamine is mono-protonated, but it is also known to form (melamineH_2_)^2+^ salts with trifluoroacetic acid (Perpétuo & Janczak, 2006), oxalic acid (Zhang *et al.*, 2005), and other strong acids. For more details and related literature see our recent review article (Aghabozorg, Manteghi *et al.*, 2008).

The formula unit of the title compound is depicted in Fig. 1. There are nine different moieties in the asymmetric unit. The cationic portion of the asymmetric unit consists of a half-hydroxonium hydrate, residing on a twofold axis, a molecule of melamineH^+^, and a half- molecule of melamineH^+^ residing on a twofold axis. The anionic portion is the [Cu(pydc)_2_]^2–^ complex ion. In addition, the asymmetric unit contains a half-molecule of neutral pydcH_2_ residing on a twofold axis, two full molecules of solvate water, a half-molecule of water on a twofold axis, and another half-molecule of water that is disordered with respect to a twofold axis. In the [Cu(pydc)_2_]^2–^, the two ligands are almost perpendicular to each other. The dihedral angle between the two pydc planes consisting of the C_7_NO_4_ set is 89.03 (3)°. The Cu—O and Cu—N distances (Table 1) are in good agreement with those seen in related Cu^II^ bis(pydc) complexes (Aghabozorg, Zabihi *et al.*, 2006, and Aghabozorg, Ghadermazi *et al.*, 2008 are two examples). Of the four nominally equivalent Cu—O distances, Cu—O5 and Cu—O7 are an average of 0.15 Å longer than the other two, indicating a weak Jahn–Teller distortion and tetragonally distorted octahedral environment. The hydroxonium hydrate (H_5_O_2_)^+^ cation that resides on a 2-fold rotation axis is bent (details are in Table 2).

A centrosymmetrically related C=O···π interaction between C=O groups and centroids of aromatic rings of pyridine-2,6-dicarboxylate is shown in Fig. 2. With regard to the overall packing, the space between layers of [Cu(pydc)_2_]^2–^ anions is filled with (melamineH)^+^ cations and pydcH_2_ molecules (Fig. 3). In fact, the layers involving the Cu^II^ complex are bridged by (melamineH)^+^ cations *via* extensive hydrogen bonds (Table 2).

The title compound is related to the recently published structure of (H_5_O_2_)(melamineH^+^)_3_[Mn^II/III^(pydc)_2_]_2_(OH)^.^(pydcH_2_) ^.^5H_2_O (Aghabozorg, Derikvand *et al.*, 2008), in which charge balance is achieved by conversion of one of the water molecules to a hydroxide. The Mn—O and Mn—N distances are longer than those of the Cu complex and there is no evidence of Jahn-Teller distortion. There are also differences in the angles about Mn that indicate a distortion away from octahedral towards tetrahedral geometry.

### Experimental

The title compound was produced by the reaction of Cu(NO_3_)_2_.6H_2_O (145 mg, 0.5 mmol), pyridine-2,6-dicarboxylic acid, pydcH_2_, (100 mg, 1 mmol) and 2,4,6-triamino-1,3,5-triazine, (melamine) (110 mg, 1 mmol) in water (50 ml). Blue crystals of the title compound were obtained by the slow evaporation of the solvent at room temperature.

### Refinement

All hydrogen atoms were initially located in a difference Fourier map. H atoms on C were refined with a riding model, C—H = 0.95 Å and U_iso(H)_ = 1.2 *U*_eq_(C). H atoms on N and O were refined with distance restraints of 0.84 (1) Å for O—H and 0.88 (1) Å for N—H. Isotropic thermal parameters were refined. The water molecule O15 is disordered with respect to a 2-fold axis and the H15*a*···H15*b* distance was refined with a distance restraint of 1.30 (2) Å. The central H atom in the [H_5_O_2_]^+^ group was freely refined with no restraints.

### Crystal data

**Table:**

(H_5_O_2_)(C_3_H_7_N_6_)_3_[Cu(C_7_H_3_NO_4_)_2_]_2_·C_7_H_5_NO_4_·6H_2_O	*F*(000) = 3048
*M**_r_* = 1481.19	*D*_x_ = 1.680 Mg m^−^^3^
Monoclinic, *C*2/*c*	Mo *K*α radiation, λ = 0.71073 Å
Hall symbol: -C 2yc	Cell parameters from 6004 reflections
*a* = 27.575 (3) Å	θ = 2.8–26.1°
*b* = 22.814 (3) Å	µ = 0.84 mm^−^^1^
*c* = 9.8068 (12) Å	*T* = 180 K
β = 108.327 (2)°	Plate, pale blue
*V* = 5856.5 (13) Å^3^	0.35 × 0.28 × 0.02 mm
*Z* = 4	

### Data collection

**Table:**

Bruker SMART APEXII diffractometer	5309 independent reflections
Radiation source: fine-focus sealed tube	3805 reflections with *I* > 2σ(*I*)
graphite	*R*_int_ = 0.051
Detector resolution: 8.3 pixels mm^-1^	θ_max_ = 25.3°, θ_min_ = 2.8°
ω scans	*h* = −33→33
Absorption correction: multi-scan (*SADABS*; Sheldrick, 1996)	*k* = −27→27
*T*_min_ = 0.758, *T*_max_ = 0.983	*l* = −11→11
26801 measured reflections	

### Refinement

**Table:**

Refinement on *F*^2^	Primary atom site location: structure-invariant direct methods
Least-squares matrix: full	Secondary atom site location: difference Fourier map
*R*[*F*^2^ > 2σ(*F*^2^)] = 0.035	Hydrogen site location: inferred from neighbouring sites
*wR*(*F*^2^) = 0.096	H atoms treated by a mixture of independent and constrained refinement
*S* = 1.03	*w* = 1/[σ^2^(*F*_o_^2^) + (0.0411*P*)^2^ + 8.7866*P*] where *P* = (*F*_o_^2^ + 2*F*_c_^2^)/3
5309 reflections	(Δ/σ)_max_ = 0.001
531 parameters	Δρ_max_ = 0.26 e Å^−^^3^
21 restraints	Δρ_min_ = −0.77 e Å^−^^3^

### Special details

**Table:**

Experimental. The crystals cracked when cooled to 90 K so the data was collected at 180 K.
Geometry. All e.s.d.'s (except the e.s.d. in the dihedral angle between two l.s. planes) are estimated using the full covariance matrix. The cell e.s.d.'s are taken into account individually in the estimation of e.s.d.'s in distances, angles and torsion angles; correlations between e.s.d.'s in cell parameters are only used when they are defined by crystal symmetry. An approximate (isotropic) treatment of cell e.s.d.'s is used for estimating e.s.d.'s involving l.s. planes.
Refinement. Refinement of *F*^2^ against ALL reflections. The weighted *R*-factor *wR* and goodness of fit *S* are based on *F*^2^, conventional *R*-factors *R* are based on *F*, with *F* set to zero for negative *F*^2^. The threshold expression of *F*^2^ > σ(*F*^2^) is used only for calculating *R*-factors(gt) *etc*. and is not relevant to the choice of reflections for refinement. *R*-factors based on *F*^2^ are statistically about twice as large as those based on *F*, and *R*- factors based on ALL data will be even larger.

### Fractional atomic coordinates and isotropic or equivalent isotropic displacement parameters (Å^2^)

**Table:**

	*x*	*y*	*z*	*U*_iso_*/*U*_eq_	Occ. (<1)
Cu1	0.157851 (13)	0.631029 (17)	−0.05095 (3)	0.03071 (12)	
O1	0.09962 (8)	0.63287 (9)	0.0509 (2)	0.0352 (5)	
O2	0.04357 (8)	0.57153 (9)	0.0993 (2)	0.0396 (5)	
O3	0.21154 (8)	0.59453 (9)	−0.1422 (2)	0.0359 (5)	
O4	0.24379 (8)	0.50682 (10)	−0.1733 (2)	0.0441 (6)	
O5	0.21596 (7)	0.65786 (9)	0.1601 (2)	0.0327 (5)	
O6	0.25253 (8)	0.73927 (9)	0.2729 (2)	0.0362 (5)	
O7	0.10282 (8)	0.65042 (10)	−0.2763 (2)	0.0374 (5)	
O8	0.07941 (8)	0.72696 (9)	−0.4265 (2)	0.0377 (5)	
N1	0.14634 (9)	0.54895 (11)	−0.0330 (2)	0.0280 (6)	
N2	0.16513 (8)	0.71590 (11)	−0.0740 (2)	0.0291 (6)	
C1	0.08130 (11)	0.58215 (13)	0.0608 (3)	0.0295 (7)	
C2	0.10898 (10)	0.53146 (13)	0.0179 (3)	0.0279 (6)	
C3	0.09751 (11)	0.47292 (14)	0.0232 (3)	0.0336 (7)	
H3	0.0712	0.4607	0.0603	0.040*	
C4	0.12506 (11)	0.43224 (15)	−0.0267 (3)	0.0377 (7)	
H4	0.1177	0.3916	−0.0245	0.045*	
C5	0.16359 (11)	0.45086 (15)	−0.0803 (3)	0.0366 (8)	
H5	0.1826	0.4233	−0.1156	0.044*	
C6	0.17368 (11)	0.50997 (14)	−0.0810 (3)	0.0296 (7)	
C7	0.21347 (11)	0.53866 (14)	−0.1368 (3)	0.0327 (7)	
C8	0.22339 (10)	0.71221 (14)	0.1673 (3)	0.0296 (7)	
C9	0.19487 (10)	0.74812 (14)	0.0362 (3)	0.0287 (7)	
C10	0.19848 (11)	0.80844 (14)	0.0269 (3)	0.0341 (7)	
H10	0.2197	0.8303	0.1056	0.041*	
C11	0.17062 (11)	0.83657 (15)	−0.0989 (3)	0.0364 (7)	
H11	0.1724	0.8779	−0.1073	0.044*	
C12	0.14016 (11)	0.80339 (14)	−0.2120 (3)	0.0333 (7)	
H12	0.1210	0.8216	−0.2993	0.040*	
C13	0.13815 (10)	0.74347 (14)	−0.1956 (3)	0.0289 (7)	
C14	0.10436 (10)	0.70318 (14)	−0.3083 (3)	0.0301 (7)	
N3	0.31880 (9)	0.69464 (11)	0.5251 (2)	0.0276 (5)	
H3A	0.3001 (9)	0.7133 (11)	0.449 (2)	0.029 (8)*	
N4	0.34173 (9)	0.60470 (11)	0.6466 (2)	0.0279 (5)	
N5	0.37566 (9)	0.69628 (11)	0.7606 (2)	0.0283 (5)	
N6	0.28551 (9)	0.60781 (12)	0.4159 (2)	0.0308 (6)	
H6A	0.2790 (12)	0.5702 (5)	0.415 (3)	0.045 (10)*	
H6B	0.2656 (10)	0.6283 (12)	0.344 (2)	0.038 (9)*	
N7	0.39782 (10)	0.60852 (12)	0.8748 (3)	0.0348 (6)	
H7A	0.4185 (10)	0.6269 (12)	0.950 (2)	0.043 (9)*	
H7B	0.3957 (13)	0.5701 (5)	0.877 (4)	0.054 (11)*	
N8	0.35019 (10)	0.78255 (12)	0.6365 (3)	0.0324 (6)	
H8A	0.3686 (9)	0.8009 (11)	0.714 (2)	0.030 (8)*	
H8B	0.3313 (11)	0.8021 (13)	0.561 (2)	0.053 (11)*	
C15	0.31528 (10)	0.63496 (13)	0.5310 (3)	0.0273 (6)	
C16	0.37112 (10)	0.63705 (13)	0.7578 (3)	0.0273 (6)	
C17	0.34873 (10)	0.72446 (13)	0.6423 (3)	0.0270 (6)	
N9	0.02898 (8)	0.83461 (10)	−0.1285 (2)	0.0271 (5)	
N10	0.0000	0.74606 (16)	−0.2500	0.0301 (8)	
H10A	0.0000	0.7074 (5)	−0.2500	0.057 (16)*	
N11	0.0000	0.91967 (15)	−0.2500	0.0308 (8)	
H11A	−0.0184 (10)	0.9405 (12)	−0.324 (2)	0.040 (9)*	
N12	0.05475 (10)	0.74580 (12)	−0.0180 (3)	0.0356 (6)	
H12A	0.0731 (10)	0.7644 (12)	0.059 (2)	0.036 (9)*	
H12B	0.0558 (13)	0.7072 (5)	−0.020 (4)	0.053 (11)*	
C18	0.0000	0.86200 (17)	−0.2500	0.0244 (8)	
C19	0.02795 (10)	0.77645 (13)	−0.1311 (3)	0.0277 (6)	
O9	0.06509 (9)	0.99585 (10)	−0.0244 (2)	0.0508 (7)	
O10	0.09675 (8)	1.07950 (9)	0.0842 (2)	0.0334 (5)	
H10B	0.1173 (12)	1.0565 (14)	0.142 (3)	0.075 (14)*	
N13	0.0000	1.05523 (15)	−0.2500	0.0279 (8)	
C20	0.0000	1.17795 (19)	−0.2500	0.0344 (10)	
H20	0.0000	1.2196	−0.2500	0.056 (15)*	
C21	0.03105 (11)	1.14704 (13)	−0.1331 (3)	0.0301 (7)	
H21	0.0522	1.1670	−0.0509	0.029 (8)*	
C22	0.03049 (10)	1.08601 (12)	−0.1391 (3)	0.0256 (6)	
C23	0.06522 (11)	1.04884 (14)	−0.0217 (3)	0.0302 (7)	
O11	0.0000	0.48788 (15)	0.2500	0.0428 (8)	
H11B	−0.0111 (14)	0.5073 (16)	0.302 (4)	0.053 (11)*	
O12	−0.01661 (12)	0.66299 (13)	0.1148 (3)	0.0631 (8)	
H12C	−0.0356 (13)	0.6839 (16)	0.049 (3)	0.079 (14)*	
H12D	0.0013 (16)	0.6374 (16)	0.092 (5)	0.100 (18)*	
H12E	0.0000	0.672 (4)	0.2500	0.17 (3)*	
O13	0.16865 (9)	1.01368 (11)	0.2655 (2)	0.0372 (5)	
H13A	0.1944 (10)	1.0045 (19)	0.242 (4)	0.080 (15)*	
H13B	0.1636 (16)	0.9829 (11)	0.306 (4)	0.079 (15)*	
O14	0.28646 (9)	0.84247 (11)	0.3914 (2)	0.0411 (6)	
H14A	0.2740 (12)	0.8119 (9)	0.347 (3)	0.043 (10)*	
H14B	0.2899 (17)	0.8674 (14)	0.333 (4)	0.088 (16)*	
O15	−0.0086 (2)	0.6313 (2)	−0.2238 (8)	0.0471 (15)	0.50
H15A	−0.006 (3)	0.599 (2)	−0.261 (12)	0.07 (3)*	0.50
H15B	−0.0385 (11)	0.630 (3)	−0.219 (8)	0.06 (3)*	0.50

### Atomic displacement parameters (Å^2^)

**Table:**

	*U*^11^	*U*^22^	*U*^33^	*U*^12^	*U*^13^	*U*^23^
Cu1	0.02553 (19)	0.0436 (3)	0.02402 (18)	0.00683 (17)	0.00929 (14)	0.00601 (16)
O1	0.0351 (11)	0.0301 (12)	0.0447 (12)	0.0009 (10)	0.0189 (10)	0.0083 (10)
O2	0.0400 (13)	0.0334 (13)	0.0575 (14)	0.0033 (10)	0.0330 (11)	0.0056 (10)
O3	0.0390 (12)	0.0375 (14)	0.0356 (11)	0.0126 (10)	0.0179 (9)	0.0096 (9)
O4	0.0431 (13)	0.0424 (14)	0.0582 (14)	0.0146 (11)	0.0324 (11)	0.0096 (11)
O5	0.0311 (11)	0.0353 (13)	0.0275 (10)	−0.0019 (10)	0.0033 (9)	0.0064 (9)
O6	0.0333 (12)	0.0361 (13)	0.0323 (11)	0.0008 (10)	0.0006 (9)	0.0084 (9)
O7	0.0388 (12)	0.0390 (14)	0.0386 (12)	0.0103 (10)	0.0181 (10)	0.0129 (10)
O8	0.0362 (12)	0.0424 (14)	0.0292 (11)	0.0005 (10)	0.0030 (9)	0.0125 (9)
N1	0.0244 (12)	0.0386 (15)	0.0204 (11)	0.0077 (11)	0.0062 (10)	0.0031 (10)
N2	0.0188 (12)	0.0456 (16)	0.0245 (12)	0.0075 (11)	0.0090 (10)	0.0114 (11)
C1	0.0289 (16)	0.0325 (18)	0.0296 (15)	0.0026 (13)	0.0131 (13)	0.0048 (13)
C2	0.0241 (14)	0.0347 (18)	0.0239 (14)	0.0027 (13)	0.0060 (12)	0.0008 (12)
C3	0.0230 (15)	0.040 (2)	0.0383 (17)	−0.0007 (14)	0.0097 (13)	−0.0012 (14)
C4	0.0326 (17)	0.0346 (19)	0.0457 (18)	0.0006 (14)	0.0119 (14)	−0.0014 (14)
C5	0.0296 (17)	0.041 (2)	0.0398 (17)	0.0075 (14)	0.0112 (14)	−0.0029 (14)
C6	0.0232 (14)	0.041 (2)	0.0233 (14)	0.0080 (14)	0.0060 (12)	0.0030 (13)
C7	0.0294 (16)	0.042 (2)	0.0271 (15)	0.0100 (15)	0.0087 (13)	0.0077 (13)
C8	0.0203 (14)	0.042 (2)	0.0261 (15)	0.0044 (13)	0.0063 (12)	0.0081 (13)
C9	0.0182 (14)	0.041 (2)	0.0282 (15)	0.0036 (13)	0.0097 (12)	0.0114 (13)
C10	0.0251 (15)	0.042 (2)	0.0348 (16)	0.0002 (14)	0.0091 (13)	0.0080 (14)
C11	0.0296 (16)	0.040 (2)	0.0422 (18)	0.0022 (15)	0.0148 (14)	0.0163 (15)
C12	0.0264 (16)	0.044 (2)	0.0307 (16)	0.0057 (14)	0.0100 (13)	0.0174 (14)
C13	0.0189 (14)	0.044 (2)	0.0267 (14)	0.0051 (13)	0.0109 (12)	0.0123 (13)
C14	0.0217 (14)	0.046 (2)	0.0256 (15)	0.0066 (14)	0.0114 (12)	0.0117 (13)
N3	0.0255 (13)	0.0310 (15)	0.0249 (12)	0.0020 (11)	0.0057 (10)	0.0106 (11)
N4	0.0280 (13)	0.0319 (14)	0.0224 (12)	0.0009 (11)	0.0061 (10)	0.0054 (10)
N5	0.0284 (13)	0.0281 (15)	0.0269 (12)	0.0022 (11)	0.0065 (10)	0.0071 (10)
N6	0.0301 (14)	0.0353 (17)	0.0244 (13)	0.0012 (12)	0.0045 (11)	0.0042 (11)
N7	0.0408 (16)	0.0286 (16)	0.0273 (14)	−0.0003 (13)	−0.0003 (12)	0.0058 (11)
N8	0.0334 (15)	0.0305 (16)	0.0298 (14)	−0.0017 (12)	0.0050 (12)	0.0063 (12)
C15	0.0227 (14)	0.0336 (18)	0.0275 (14)	0.0021 (13)	0.0106 (11)	0.0055 (13)
C16	0.0252 (14)	0.0330 (18)	0.0241 (14)	0.0008 (13)	0.0084 (11)	0.0053 (12)
C17	0.0226 (14)	0.0321 (18)	0.0288 (15)	0.0009 (13)	0.0118 (12)	0.0059 (12)
N9	0.0284 (13)	0.0208 (14)	0.0283 (12)	0.0012 (10)	0.0035 (10)	0.0026 (10)
N10	0.0279 (19)	0.021 (2)	0.038 (2)	0.000	0.0060 (15)	0.000
N11	0.036 (2)	0.021 (2)	0.0269 (19)	0.000	−0.0024 (16)	0.000
N12	0.0334 (15)	0.0259 (17)	0.0409 (16)	0.0012 (12)	0.0024 (12)	0.0097 (13)
C18	0.0213 (19)	0.022 (2)	0.027 (2)	0.000	0.0041 (16)	0.000
C19	0.0234 (14)	0.0267 (18)	0.0336 (16)	0.0009 (12)	0.0097 (12)	0.0025 (12)
O9	0.0626 (16)	0.0264 (14)	0.0390 (13)	−0.0015 (11)	−0.0189 (11)	0.0011 (10)
O10	0.0322 (12)	0.0327 (13)	0.0281 (11)	−0.0022 (10)	−0.0008 (9)	−0.0058 (9)
N13	0.0258 (18)	0.029 (2)	0.0262 (17)	0.000	0.0044 (14)	0.000
C20	0.031 (2)	0.024 (3)	0.046 (3)	0.000	0.010 (2)	0.000
C21	0.0267 (15)	0.0289 (18)	0.0340 (16)	−0.0041 (13)	0.0086 (13)	−0.0063 (12)
C22	0.0241 (15)	0.0244 (17)	0.0271 (14)	−0.0020 (12)	0.0064 (12)	−0.0028 (11)
C23	0.0283 (16)	0.033 (2)	0.0260 (15)	−0.0018 (13)	0.0043 (12)	−0.0054 (12)
O11	0.062 (2)	0.030 (2)	0.042 (2)	0.000	0.0255 (18)	0.000
O12	0.087 (2)	0.069 (2)	0.0308 (14)	0.0485 (17)	0.0157 (14)	0.0130 (13)
O13	0.0320 (13)	0.0404 (15)	0.0374 (12)	0.0012 (11)	0.0086 (10)	0.0076 (10)
O14	0.0568 (15)	0.0361 (15)	0.0267 (12)	−0.0032 (12)	0.0077 (11)	0.0052 (11)
O15	0.032 (4)	0.027 (3)	0.090 (5)	0.000 (2)	0.030 (3)	0.003 (3)

### Geometric parameters (Å, °)

**Table:**

Cu1—N1	1.916 (3)	N5—C16	1.357 (4)
Cu1—N2	1.967 (3)	N6—C15	1.322 (4)
Cu1—O3	2.126 (2)	N6—H6A	0.876 (13)
Cu1—O1	2.142 (2)	N6—H6B	0.88 (3)
Cu1—O5	2.2670 (19)	N7—C16	1.323 (4)
Cu1—O7	2.296 (2)	N7—H7A	0.88 (3)
O1—C1	1.279 (3)	N7—H7B	0.879 (12)
O2—C1	1.237 (3)	N8—C17	1.328 (4)
O3—C7	1.276 (4)	N8—H8A	0.88 (3)
O4—C7	1.242 (3)	N8—H8B	0.88 (3)
O5—C8	1.255 (4)	N9—C19	1.327 (4)
O6—C8	1.256 (3)	N9—C18	1.360 (3)
O7—C14	1.248 (4)	N10—C19	1.367 (3)
O8—C14	1.269 (3)	N10—C19^i^	1.367 (3)
N1—C2	1.339 (4)	N10—H10A	0.882 (12)
N1—C6	1.343 (4)	N11—C18	1.315 (5)
N2—C13	1.347 (3)	N11—H11A	0.88 (2)
N2—C9	1.350 (4)	N12—C19	1.323 (4)
C1—C2	1.516 (4)	N12—H12A	0.87 (3)
C2—C3	1.377 (4)	N12—H12B	0.876 (13)
C3—C4	1.383 (4)	C18—N9^i^	1.360 (3)
C3—H3	0.9500	O9—C23	1.209 (4)
C4—C5	1.391 (4)	O10—C23	1.325 (3)
C4—H4	0.9500	O10—H10B	0.85 (3)
C5—C6	1.377 (4)	N13—C22	1.345 (3)
C5—H5	0.9500	N13—C22^i^	1.345 (3)
C6—C7	1.520 (4)	C20—C21^i^	1.388 (4)
C8—C9	1.519 (4)	C20—C21	1.388 (4)
C9—C10	1.385 (4)	C20—H20	0.9500
C10—C11	1.388 (4)	C21—C22	1.393 (4)
C10—H10	0.9500	C21—H21	0.9500
C11—C12	1.387 (4)	C22—C23	1.506 (4)
C11—H11	0.9500	O11—H11B	0.81 (3)
C12—C13	1.379 (4)	O12—H12C	0.84 (3)
C12—H12	0.9500	O12—H12D	0.84 (3)
C13—C14	1.512 (4)	O12—H12E	1.274 (14)
N3—C15	1.367 (4)	O13—H13A	0.84 (3)
N3—C17	1.368 (4)	O13—H13B	0.84 (3)
N3—H3A	0.874 (10)	O14—H14A	0.84 (3)
N4—C15	1.332 (3)	O14—H14B	0.84 (3)
N4—C16	1.355 (4)	O15—H15A	0.84 (3)
N5—C17	1.329 (3)	O15—H15B	0.84 (3)
			
N1—Cu1—N2	176.51 (9)	N2—C13—C14	113.9 (3)
N1—Cu1—O3	79.24 (9)	C12—C13—C14	123.7 (2)
N2—Cu1—O3	103.15 (9)	O7—C14—O8	126.2 (3)
N1—Cu1—O1	78.83 (9)	O7—C14—C13	117.7 (2)
N2—Cu1—O1	98.79 (9)	O8—C14—C13	116.1 (3)
O3—Cu1—O1	158.06 (8)	C15—N3—C17	119.5 (2)
N1—Cu1—O5	105.67 (8)	C15—N3—H3A	119.4 (19)
N2—Cu1—O5	76.75 (8)	C17—N3—H3A	121.0 (19)
O3—Cu1—O5	95.97 (8)	C15—N4—C16	115.6 (3)
O1—Cu1—O5	89.28 (8)	C17—N5—C16	116.2 (2)
N1—Cu1—O7	101.42 (9)	C15—N6—H6A	123 (2)
N2—Cu1—O7	76.13 (8)	C15—N6—H6B	120 (2)
O3—Cu1—O7	90.42 (7)	H6A—N6—H6B	116 (3)
O1—Cu1—O7	94.57 (7)	C16—N7—H7A	122 (2)
O5—Cu1—O7	152.87 (8)	C16—N7—H7B	120 (2)
C1—O1—Cu1	112.94 (18)	H7A—N7—H7B	119 (3)
C7—O3—Cu1	113.53 (18)	C17—N8—H8A	117 (2)
C8—O5—Cu1	111.97 (16)	C17—N8—H8B	122 (2)
C14—O7—Cu1	111.32 (18)	H8A—N8—H8B	121 (3)
C2—N1—C6	121.0 (3)	N6—C15—N4	120.7 (3)
C2—N1—Cu1	119.6 (2)	N6—C15—N3	117.6 (2)
C6—N1—Cu1	119.2 (2)	N4—C15—N3	121.7 (3)
C13—N2—C9	118.8 (3)	N7—C16—N4	117.3 (3)
C13—N2—Cu1	120.9 (2)	N7—C16—N5	116.8 (3)
C9—N2—Cu1	120.15 (18)	N4—C16—N5	125.9 (2)
O2—C1—O1	126.2 (3)	N8—C17—N5	120.3 (3)
O2—C1—C2	118.7 (3)	N8—C17—N3	118.6 (2)
O1—C1—C2	115.1 (3)	N5—C17—N3	121.1 (3)
N1—C2—C3	121.0 (3)	C19—N9—C18	116.1 (2)
N1—C2—C1	112.9 (3)	C19—N10—C19^i^	119.1 (4)
C3—C2—C1	126.0 (3)	C19—N10—H10A	120.47 (18)
C2—C3—C4	118.7 (3)	C19^i^—N10—H10A	120.47 (18)
C2—C3—H3	120.7	C18—N11—H11A	123 (2)
C4—C3—H3	120.7	C19—N12—H12A	119 (2)
C3—C4—C5	119.9 (3)	C19—N12—H12B	121 (2)
C3—C4—H4	120.1	H12A—N12—H12B	120 (3)
C5—C4—H4	120.1	N11—C18—N9^i^	117.36 (18)
C6—C5—C4	118.7 (3)	N11—C18—N9	117.36 (18)
C6—C5—H5	120.6	N9^i^—C18—N9	125.3 (4)
C4—C5—H5	120.6	N12—C19—N9	120.6 (3)
N1—C6—C5	120.7 (3)	N12—C19—N10	117.6 (3)
N1—C6—C7	112.9 (3)	N9—C19—N10	121.7 (3)
C5—C6—C7	126.4 (3)	C23—O10—H10B	110 (3)
O4—C7—O3	126.5 (3)	C22—N13—C22^i^	117.0 (3)
O4—C7—C6	118.7 (3)	C21^i^—C20—C21	118.9 (4)
O3—C7—C6	114.8 (3)	C21^i^—C20—H20	120.5
O5—C8—O6	125.7 (3)	C21—C20—H20	120.5
O5—C8—C9	117.0 (3)	C20—C21—C22	118.5 (3)
O6—C8—C9	117.4 (3)	C20—C21—H21	120.7
N2—C9—C10	121.8 (3)	C22—C21—H21	120.7
N2—C9—C8	114.0 (3)	N13—C22—C21	123.5 (3)
C10—C9—C8	124.3 (3)	N13—C22—C23	114.2 (3)
C9—C10—C11	119.1 (3)	C21—C22—C23	122.3 (2)
C9—C10—H10	120.4	O9—C23—O10	122.8 (3)
C11—C10—H10	120.4	O9—C23—C22	123.3 (2)
C12—C11—C10	119.0 (3)	O10—C23—C22	113.9 (3)
C12—C11—H11	120.5	H12C—O12—H12D	118 (4)
C10—C11—H11	120.5	H12C—O12—H12E	130 (4)
C13—C12—C11	119.0 (3)	H12D—O12—H12E	109 (4)
C13—C12—H12	120.5	H13A—O13—H13B	102 (4)
C11—C12—H12	120.5	H14A—O14—H14B	109 (4)
N2—C13—C12	122.3 (3)	H15A—O15—H15B	102 (3)

Symmetry codes: (i) −*x*, *y*, −*z*−1/2.

### Hydrogen-bond geometry (Å, °)

**Table:**

*D*—H···*A*	*D*—H	H···*A*	*D*···*A*	*D*—H···*A*
N3—H3A···O6	0.87 (3)	1.90 (1)	2.766 (3)	168 (3)
N6—H6A···O4^ii^	0.88 (1)	2.06 (2)	2.880 (4)	155 (3)
N6—H6B···O5	0.88 (3)	2.01 (1)	2.872 (3)	167 (3)
N7—H7A···N9^iii^	0.88 (3)	2.09 (1)	2.965 (3)	176 (3)
N7—H7B···O9^iii^	0.88 (1)	2.13 (3)	2.815 (3)	134 (3)
N8—H8B···O14	0.88 (3)	1.96 (1)	2.842 (3)	177 (3)
N10—H10A···O15	0.88 (1)	1.78 (1)	2.649 (6)	167 (2)
N11—H11A···O9^i^	0.88 (3)	2.06 (1)	2.939 (3)	172 (3)
N12—H12A···N5^iii^	0.87 (3)	2.09 (1)	2.959 (4)	173 (3)
N12—H12B···O1	0.88 (1)	2.07 (2)	2.846 (3)	146 (3)
O10—H10B···O13	0.85 (3)	1.83 (3)	2.671 (3)	172 (4)
O11—H11B···O2^iv^	0.81 (3)	2.10 (3)	2.896 (3)	167 (4)
O12—H12C···O8^i^	0.84 (3)	1.72 (3)	2.555 (3)	173 (4)
O12—H12D···O2	0.84 (3)	1.89 (4)	2.700 (3)	162 (5)
O12—H12E···O12^iv^	1.27 (1)	1.27 (1)	2.518 (5)	162 (8)
O13—H13A···O4^v^	0.84 (3)	2.04 (3)	2.870 (3)	172 (4)
O13—H13B···N4^iii^	0.84 (3)	2.07 (2)	2.876 (3)	162 (4)
O14—H14A···O6	0.84 (3)	1.83 (1)	2.662 (3)	172 (3)
O14—H14B···O3^v^	0.84 (3)	2.05 (4)	2.850 (3)	160 (4)
O15—H15A···O11^vi^	0.84 (3)	1.98 (6)	2.748 (6)	151 (11)
O15—H15B···O7^i^	0.84 (3)	1.82 (2)	2.636 (5)	163 (7)

Symmetry codes: (ii) *x*, −*y*+1, *z*+1/2; (iii) −*x*+1/2, −*y*+3/2, −*z*+1; (i) −*x*, *y*, −*z*−1/2; (iv) −*x*, *y*, −*z*+1/2; (v) −*x*+1/2, −*y*+3/2, −*z*; (vi) −*x*, −*y*+1, −*z*.
